# A Rail Fastener Tightness Detection Approach Using Multi-source Visual Sensor

**DOI:** 10.3390/s20051367

**Published:** 2020-03-02

**Authors:** Qiang Han, Shengchun Wang, Yue Fang, Le Wang, Xinyu Du, Hailang Li, QiXin He, Qibo Feng

**Affiliations:** 1 School of Science, Beijing Jiaotong University, Beijing 100044, China; chivalry@rails.cn (Q.H.); heqixin@bjtu.edu.cn (Q.H.); 2 Infrastructure Inspection Research Institute, China Academy of Railway Sciences Corporation Limited, Beijing 100081, China; wangshengchun@rails.com (S.W.); fangyue@rails.cn (Y.F.); 15121630@bjtu.edu.cn (L.W.); lhldh@rails.cn (H.L.)

**Keywords:** railway defect detection, rail fastener, tightness detection, structured light sensor, multi-source visual data, object detection, image segmentation

## Abstract

At present, the method of two-dimensional image recognition is mainly used to detect the abnormal fastener in the rail-track inspection system. However, the too-tight-or-too-loose fastener condition may cause the clip of the fastener to break or loose due to the high frequency vibration shock, which is difficult to detect from the two-dimensional image. In this practical application background, 3D visual detection technology provides a feasible solution. In this paper, we propose a fundamental multi-source visual data detection method, as well as an accurate and robust fastener location and nut or bolt segmentation algorithm. By combining two-dimensional intensity information and three-dimensional depth information generated by the projection of line structural light, the locating of nut or bolt position and accurate perception of height information can be realized in the dynamic running environment of railway. The experimental results show that the static measurement accuracy in the vertical direction using the structural light vision sensor is 0.1 mm under the laboratory condition, and the dynamic measurement accuracy is 0.5 mm under the dynamic train running environment. We use dynamic template matching algorithm to locate fasteners from 2D intensity map, which achieves 99.4% accuracy, then use the watershed algorithm to segment the nut and bolt from the corresponding depth image of located fastener. Finally, the 3D shape of the nut and bolt is analyzed to determine whether the nut or bolt height meets the local statistical threshold requirements, so as to detect the hidden danger of railway transportation caused by too loose or too tight fasteners.

## 1. Introduction

The rail fastener is an important infrastructure for railway tracks that are used to secure rails to sleepers or concrete foundation bed. However, due to the large span of the distribution of the railway infrastructure, changing environment, and the impact of the train vibration during the daily running will loosen or break the fasteners, which will further lead to the loss or damage of the fasteners, which will seriously affect the train running safety, or even cause a train derailment accident [1,2]. In the past, relying on the method of manual patrol, the inspection workload was large, the visual conditions at night were poor, and the inspection work was inefficient. At the same time, it may bring unknown security risks to the patrol personnel. This backward manual inspection method consumes a large amount of money, manpower, and resources due to its time-consuming nature, inefficiency, subjectivity, and other shortcomings, which can no longer meet the high-efficiency and accurate requirements of modern railway inspection, so the new requirements were put forward to develop more efficient and automated infrastructure inspection system.

Under such circumstances, the track condition inspection system based on train-borne imaging equipment has been widely developed and used by various countries. For example, Australia developed track scanning system (RAILSCAN) to capture the full-line video, and then perform the late offline video playback to find the fault; Germany’s track inspection system (RAILCHECK) uses high-definition digital imaging and image processing technology to achieve automatic detection of track structure abnormalities. The US developed the visual inspection system (TVIS), which can comprehensively detect defects on the surface of the rail head, abnormal condition of the fastener, and damage of the track bed; Italy and other countries have also developed track inspection equipment to achieve the automatic identification of anomalies, including rail surface defects and fasteners. After the opening of the Qinghai-Tibet Railway in China, in order to cope with the harsh plateau climate, the track inspection system was first introduced from abroad in 2007 to replace the manual inspection of the line. The China Academy of Railway Sciences developed a track inspection system based on computer vision in 2013 successfully [3,4,5,6,7,8,9,10,11].

As one of the important application fields of computer vision, visual inspection technology is based on modern optics, integrating modern science and technology such as computer technology, laser technology, image processing, and analysis technology to constitute an integrated measurement system. Visual inspection technology can be divided into two types: Two-dimensional detection and three-dimensional detection. Rail track detection based on two-dimensional image processing is a classic and important technology in many detection technologies, and have been widely used in the field of rail track anomaly detection. Trosino et al. [12] first introduced computer vision technology to the railway field to assist in the manual detection of track equipment. The system only realizes the collection and storage of track images, which is convenient for subsequent manual browsing, but does not have automatic analysis capability. With the development of image processing, pattern recognition, and computer vision technology, various intelligent analysis systems for automatic detection have been introduced. Detection models based on artificial design features and machine learning have been applied to all aspects of track inspection. This kind of scheme draws on the research methods of transplanting “face recognition” and “pedestrian detection” and other classic problems. Some representative ones are: Xie et al. [13,14,15] designed the fastener identification system using the framework of Haar feature and Adaboost classifier in “Face Recognition”, and Li, Hang, Liu, Dong et al. [16,17,18,19,20] used the HOG feature adopted in “Pedestrian Detection” to describe the fastener, and the pattern classification system is designed in combination with the K-Nearest Classifier (KNN) and Support Vector Machine (SVM) theory. Dou et al. [21,22] proposed a method based on contrast transformation to identify the surface defect of the rail head, and to identify the fastener anomalies by dynamically updating the fastener template library with the KNN dynamic clustering framework. Limited by the artificial feature-based modeling method, the feature representation ability of the fastener image is insufficient. The above methods all have problems such as insufficient generalization for different lines and insufficient recall factor in the open environment. In response to these problems, some researchers have begun to use the deep learning neural network for intelligent analysis of track images. Faghih-Roohi et al. [23] used convolutional neural networks to locate and identify rail head surface defects. Liu [24] proposed a railway foreign object location and detection method based on Deep Trust Network (DBN) for railway foreign body intrusion. Zhao [25] proposed a fastener condition detection method based on the Siamese deep network model, whose input is a pair of images, and the fastener image features are extracted by determining the similarity of the pair of images. Gilbert et al. [26] has applied the convolution neural network (CNN) to fastener recognition, which proposes a full convolution neural network structure constructed based on the multitasking learning framework.

Known from above research, the visual detection technology based on 2D visual sensor has been researched and developed for decades in railway infrastructure inspection, and has technically overcome the influence of environmental interference and other factors. However, the maximum limitation of the three-dimensional information loss in the two-dimensional image restricts the further development of the detection technology. After decades of multidisciplinary research, modern hardware and algorithms are contributing to the continuous advancement of 3D scanning technology. These techniques allow digital capture of the natural surface geometry of objects. Using 3D scanning techniques based on structured light, projecting different patterns or structures into the scanned scene, the deformation of the original structure on the scanned scene can be used to restore the geometry of the scene. The current structured light technology can capture the 3D geometry of the scene at a very high sampling frequency. In view of the above advantages, the application of 3D scanning technology based on structured light is more and more extensive. The increasingly improved 3D imaging method has gradually become a hotspot in many application fields.

Safa et al. [27] introduced a three-dimensional imaging-based correlation analysis method for dense point cloud information on rail corrosion damage; Zhan et al. [28] outlines the research progress in three-dimensional dynamic detection of tunnels a railway based on multi-camera and structured light vision systems; Zhou et al. [29] proposed a three dimensional point cloud tunnel crack detection method based on mobile laser scanning system (MLS) with an accuracy of 0.03 m, which can effectively calculate the differences among various crack types; Rikhotso et al. [30] proposed a method for acquiring rail condition and detecting surface defects using 3D image acquisition and modeling; Gabara et al. [31] proposed an image-based point cloud measurement method for the determination of geometrical parameters of railway tracks; Xiong et al. [32] proposed a new type of three-dimensional laser profiling system, which acquires the rail surface profile data using laser scanners, odometers, inertial measurement units (IMUs), and global positioning systems (GPS), and combines all measurement profiles to form a rail surface. Yi et al. [33] proposed a new profile registration method for sparse iterative nearest neighbors for 3D track measurements, and introduced the distance between the Hausdorff distance measurement wear model and the reference model; Mao et al. [34] proposed a high-speed railway fastener detection method based on structured light sensor, which uses the structured light sensor to obtain high-precision, high-density fastener point cloud, and uses the decision tree classifier to defect the fastener and carry out the detailed classification; Cui et al. [35] proposed a real-time measurement system for high-speed railway fastener geometric parameters based on 3D laser profilometer, which obtained a dense and accurate 3D point cloud of high-speed rail fasteners. This system can replace manual measurement, greatly improving the efficiency of fastener geometry measurement. 

The fastener is the intermediate component for joining the rail with the sleeper, fixes the rail on the sleeper tightly by fastening the clip to the bottom of the rail, maintains the gauge, and prevents the longitudinal and lateral movement of the rail relative to the sleeper. For the abnormal condition of the rail fastener, the root cause of the abnormality of the fastener is the high-frequency vibration of the fastener system caused by the lateral impact of the rail during the running of the train. If the bolt is tightened too tightly, it may cause the clip to break. Otherwise, the buckle pressure will be invalid, and the clip will be displaced and lost if too loose. Therefore, many clip failures are caused by the continuing development of abnormal bolt. It is crucial to detect the condition of the bolt of the fasteners, so that the hidden danger can be found in the incipient stage of the fastener defect, thereby preventing the failure from further development. However, most of the existing researches focus on the automatic detection of the appearance condition of the clip based on the two-dimensional visible image, which can accurately recognize the change of the appearance shape such as the loss, breakage, and deformation of the clip, and are rarely involved for the abnormal condition detection of the bolt or nut. In addition, the over-tightening or too looseness of the bolt visually reflects the displacement change in the depth direction, and there is no obvious shape and position change in the two-dimensional plane of the image. It is difficult to find such subtle changes in the depth direction based on the detection technique of the two-dimensional visible image.

At present, it is not uncommon for the track condition detection method based on two-dimensional visible light image or three-dimensional point cloud data, which have different detection effects between different methods. The two-dimensional image can directly reflect the geometric shape of the object and the change of the spatial position, and has achieved good results in locating the fastener position from the whole track image, and it’s more accurate and robust to segment the bolt from the located fastener sub-region. Therefore, it is a feasible solution to improve the accuracy of the bolt condition detection to comprehensively detect the location and height of the bolt by multiple types of data sources. In view of the respective advantages of two-dimensional intensity information and three-dimensional depth information in target detection and segmentation, the two types of data generated by the line structure light sensor are organically combined to realize the location of the bolt position and accurate perception of the bolt height. First, the position of the fastener is determined in the two-dimensional intensity map, and the bolt sub-region is found in the three-dimensional depth map of the track according to the result of the fastener positioning; further, according to the geometry prior and spatial position prior of the fastener structure and distribution, as well as relevant railway standard, we accurately locate the position of the bolt in the bolt sub-region; finally, we analyze the 3D shape feature of the bolt, determine whether the height of the bolt meets the requirements, and thus detect and find the safety hazard of railway running caused by the bolt failure.

The remaining chapters of this paper are organized as follows: Section 2 introduces the types of common fasteners and the fundamentals of fastener tightness detection. The detection model and implementation method are detailed in Section 3. Section 4 verifies the static and dynamic measurement precision of the sensor, and carries out a full experimental demonstration of the detection effect. Finally, the work of the full paper is summarized, and conclusions and suggestions are given.

## 2. Fundamentals

**Figure 1:**
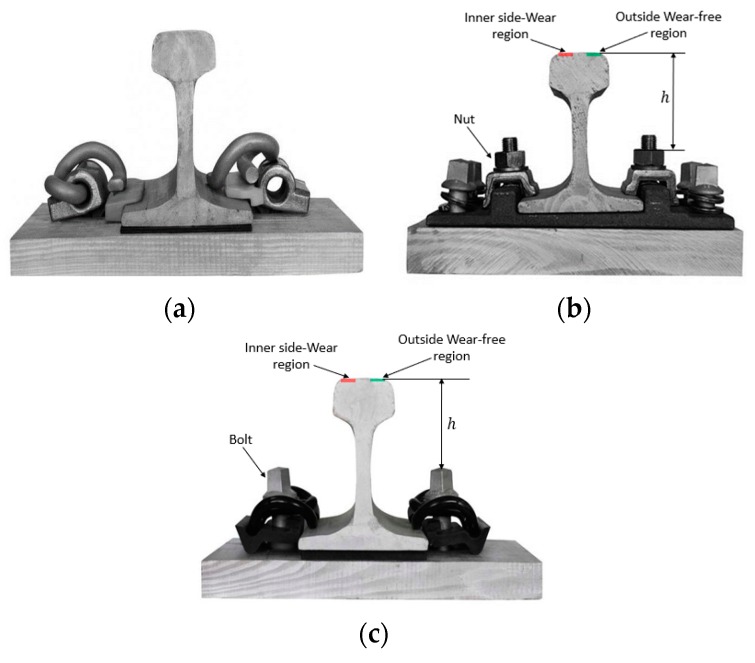
Rail fastener types: (**a**) Bolt-free fastener; (**b**) nut-fastening fastener; (**c**) bolt-fastening fastener.

Figure 1 shows some examples of the main types of rail fasteners, which can be divided into bolt-free fastener, nut-fastening fastener, and bolt-fastening fastener according to the fixing method of the fastener. The bolt-free fasteners are only used on passenger dedicated lines, and nut-fastening fasteners are widely used in ballast track. Bolt-fastening fasteners are common in high-speed ballast-less track, which are a newly developed fastener structure in recent years. For bolt-free fasteners, the number of such fasteners is small throughout railway lines, and the structure of the fasteners will inevitably change the clip position once they are loose, so the traditional two-dimensional image sensors can detect such abnormalities. The research in this paper is mainly aimed at fastener structures with nuts or bolts, that is, the types of clips fastened by nuts for the ballast track and fastened by bolts for the ballast-less track.

Fastener detection is an extremely important task in railway operation safety inspection. For fastener detection, a very important task is accurate target detection, and positioning the position of the nut or bolt and determining whether it is too loose or too tight. For the types of fasteners shown in Figure 1b,c, due to the effects of construction conditions, the impact of lateral rail forces, wheel-rail resonance, etc., the bolts or nuts will inevitably become too tight or loose. At present, there are few related researches on fastener tightness detection. Traditional vision sensor-based methods are difficult to achieve this purpose. The most representative is the three-dimensional point cloud data obtained by structured light sensor proposed by Mao et al. [34], and the tightness of the fastener is reflected by detecting the buckling deformation of the clip. However, this method requires fine segmentation of the clip area, and the clip area is extremely susceptible to foreign object occlusion and light changes. Therefore, this method has weak anti-interference ability and is not easy to generalize and apply. In addition, for some fastener types, as shown in Figure 1b, there is no clip structure. Therefore, in order to accurately sense the position and height of a nut or bolt in a fastener, a 3D sensor needs to be used.

**Figure 2:**
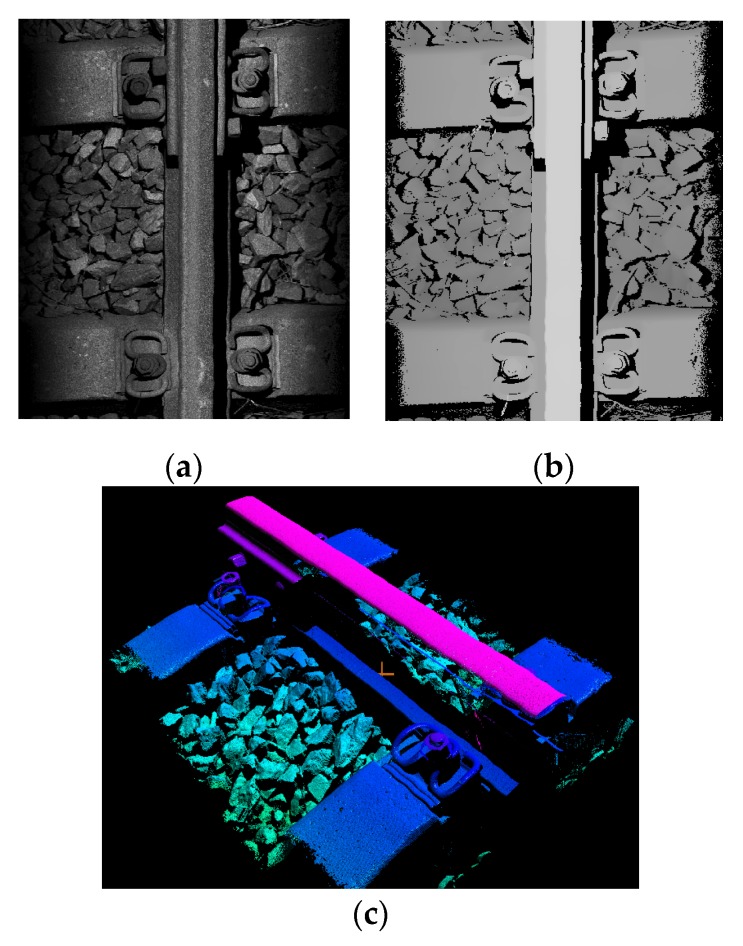
Nut looseness: (**a**) Intensity map; (**b**) depth map; (**c**) point cloud 3D reconstruction.

In this paper, we introduce a new type of structured light sensor to locate the position of the bolt and nut in the fastener and detect its height to determine if there is a potential safety hazard. As shown in Figure 2, the sensor can both capture 2D intensity map *I(x, y)* and 3D depth map *D(x, y)* from structured light sensor (*x, y* is the pixel coordinates of mapped image). It can be observed from the figure that the loosening of the fastener is difficult to identify in the 2D intensity map and the magnitude of the loosening cannot be known. But from the three-dimensional depth map, it is easy to know the position difference between the nut and the bolt (The depth value of upper left loosening nut is 24.87 mm, and the lower left normal nut is 7.22 mm), so that the loosening degree of the fastener can be quantitatively evaluated. Therefore, three-dimensional information provides powerful data support for anomaly detection and quantitative analysis, and 2D–3D data fusion analysis will definitely improve the system’s detection capability.

The corresponding algorithm implementation process includes the following three processes: (1) Positioning the rails and fasteners, (2) positioning the nut or bolt, (3) use the depth information to determine the altitude difference of the nut or bolt relative to the height of rail surface. Because the inside of the rail is the wheel-rail contact surface, there is unavoidably a lot of uncertain wear values, so the outside wear-free area of rail surface is used as a comparison basis.

## 3. Methodology

### 3.1. Fastener Tightness Detection Model

**Figure 3:**
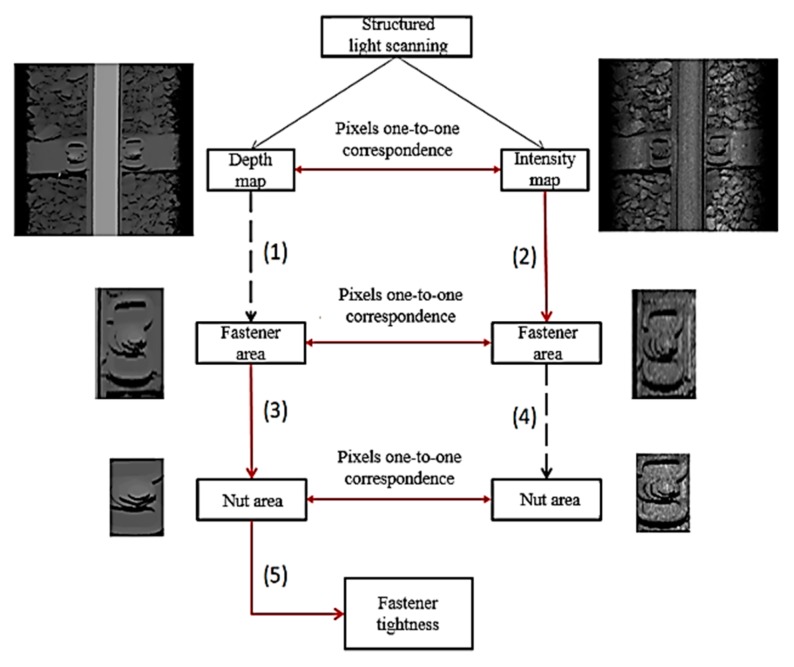
Method for detecting nut position based on 2D/3D map.

Through the structured light scanning device (Ranger3 3D Camera by SICK company, based in Waldkirch (Breisgau), Germany), a depth map and an intensity map can be obtained at the same time. The pixels of these two maps correspond one-to-one, that is, corresponding image regions in the intensity map appear at the same position in the depth map. If a certain target is detected in the intensity map, the corresponding target can be found at the same position in the depth map, and vice versa. Therefore, the nut or bolt can be located in the depth map and the intensity map, respectively, that is, the location of fasteners and nuts or bolts are searched for in the depth map and the intensity map, respectively. As shown in the Figure 3, the method for detecting nut position based on 2D intensity map *I(x, y)* and 3D depth *D(x, y)* map can be described as below:

(1) Perform fastener positioning on the depth map to obtain the fastener region, which is formulated as
  (1) $$pos\left(D\left(x,y\right)\right)\to \overset{I_{s}}{\underset{x=I_{b}}{\cup }}\overset{J_{s}}{\underset{y=J_{b}}{\cup }}D\left(x,y\right)$$
  where pos() denotes fastener positioning algorithm; $\left[I_{b},I_{s}\right]$ and $\left[J_{b},J_{s}\right]$ denote the pixel coordinate range of detection result (fastener region); ∪ denotes the union operation for the pixels.
(2) Perform fastener positioning on the intensity map to obtain the fastener region, which is formulated as
  (2) $$pos\left(I\left(x,y\right)\right)\to \overset{I_{s}}{\underset{x=I_{b}}{\cup }}\overset{J_{s}}{\underset{y=J_{b}}{\cup }}I\left(x,y\right)$$
(3) Segment the nut and bolt sub-region in the detected fastener region from depth map, which is formulated as
  (3) $$seg\left(\overset{I_{s}}{\underset{x=I_{b}}{\cup }}\overset{J_{s}}{\underset{y=J_{b}}{\cup }}D\left(x,y\right)\right)\to \overset{N_{dep}}{\underset{n=1}{\cup }}\overset{I_{s}^{\prime }}{\underset{x=I_{b}^{\prime }}{\cup }}\overset{J_{s}^{\prime }}{\underset{y=J_{b}^{\prime }}{\cup }}D^{\prime }\left(n,x,y\right)$$
  where seg() denotes nut and bolt segmentation algorithm; *N_dep_* is the number of the segmented sub-regions from depth map; $D^{\prime }\left(n,x,y\right)$ denotes *n*^th^ single sub-region of the segmented results from depth map, which meet the condition $D^{\prime }\left(n,x,y\right)\subseteq D\left(x,y\right)$; $\left[I_{b}^{\prime },I_{s}^{\prime }\right]$ and $\left[J_{b}^{\prime },J_{s}^{\prime }\right]$ denote the pixel coordinate range of segmentation result (nut and bolt sub-region), which meet the condition $\left[I_{b}^{\prime },I_{s}^{\prime }]\subset [I_{b},I_{s}\right]$.
(4) Segment nut and bolt sub-region in the detected fastener region from intensity map, which is formulated as
  (4) $$seg\left(\overset{I_{s}}{\underset{x=I_{b}}{\cup }}\overset{J_{s}}{\underset{y=J_{b}}{\cup }}I\left(x,y\right)\right)\to \overset{N_{int}}{\underset{n=1}{\cup }}\overset{I_{s}^{\prime }}{\underset{x=I_{b}^{\prime }}{\cup }}\overset{J_{s}^{\prime }}{\underset{y=J_{b}^{\prime }}{\cup }}I^{\prime }\left(n,x,y\right)$$
  where *N_int_* is the number of the segmented sub-regions from intensity map; $I^{\prime }\left(n,x,y\right)$ denotes *n*th single sub-region of the segmented results from intensity map, which meet the condition $I^{\prime }\left(n,x,y\right)\subseteq I\left(x,y\right)$.
(5) Analyze geometric features in the nut and bolt sub-region to determine the condition of nut and bolt from depth map, which is formulated as
  (5) $$any\left(\overset{N_{dep}}{\underset{n=1}{\cup }}\overset{I_{s}^{\prime }}{\underset{x=I_{b}^{\prime }}{\cup }}\overset{J_{s}^{\prime }}{\underset{y=J_{b}^{\prime }}{\cup }}D^{\prime }\left(n,x,y\right)\right)\to \left(tightness,\;looseness\right)$$
  where any() denotes the feature analysis algorithm; (tightness, looseness) is a binary vector and denotes the final detection result.

The black dotted arrows in the figure indicate the methods with poor experimental results. The basic flow we adopted is: (2) → (3) → (5), and the subsequent experimental results prove that the effect of process (1) and process (4) is not good while stable and reasonable test results cannot be obtained. The main reason is determined by the characteristics of the depth map and intensity map:

- Intensity map: The texture information is rich, and the area smoothing features are not obvious.
- Depth map: The region smoothing feature is obvious, and the texture information is not rich.

The rail area is a large detection target and has significant intensity and depth characteristics. The method of feature projection analysis is easy to locate in the rail area. The area of the fastener is relatively small, and the area is about 1% of the area of the image area. That is to say, the detection of the fastener is a small target detection and requires rich texture information. Therefore, the detection result of the fastener in the intensity map is much better than that in the depth map. Once you find the fastener, you need to find the nut and bolt further in the fastener area. First, the area of the image area of the nut (bolt) in the fastener area is larger, about 15%. Second, the nut (bolt) appears near the center of the fastener area. Therefore, the detection of nuts (bolts) is suitable to be realized by the method of region segmentation. For region segmentation, sequential images with smooth regions will achieve good segmentation results, while images with rich texture features often fail to achieve good segmentation results. Therefore, the positioning result of the nut (bolt) in the depth map (fastener area) is much better than the positioning result of the nut (bolt) in the intensity map (fastener area). It should be noted that, because the pixels of the depth map and the intensity map correspond one-to-one, while the fastener is detected in the intensity map, the corresponding fastener is also positioned in the depth map. Therefore, the basic process of positioning the nut (bolt) is (2) → (3) in Figure 3, that is, detecting the fastener in the intensity map, and then positioning the nut (bolt) in the same image area in the depth map). After successfully positioning the nut (bolt), you can further determine the condition of the nut (bolt) (that is, (5) in Figure 3) based on the height difference between the nut (bolt) and the surface of the rail.

Below we will detail the basic methods of fastener positioning and nut or bolt detection.

### 3.2. Locating Rail Fastener Area based on Online Learning Strategy

• Rail fastener region positioning

In order to make full use of the prior information of the spatial distribution of fasteners, the analyzed track image should contain multiple pairs of fasteners. In this paper, the spatial sampling distance of each track image is about 2 m. Each track image contains at least 6 fastener regions, and there is no extreme case where all the fasteners in the track image are lost. Therefore, by using the sliding window method and the template library, at least one fastener area can be located in the candidate area of rail fasteners. Then, other rail fastener areas can be further inferred based on the longitudinal and lateral installation intervals between the rail fasteners. The construction method of the template library will be described in detail below.

The track positioning method proposed in this section is divided into three steps:

Step 1: For the 90×100 size of the fastener area as a window, use the sliding window method to extract sub-windows in a fixed step within the track fastener candidate area, and then calculate the similarity between the sub-window and each template in the template library.

The key problem in measuring the similarity of two images is to find suitable feature descriptors and similarity measurement methods. From the characteristics of the track fastener area, its shape and texture features are quite different from other areas. Therefore, texture features can be extracted to represent the fastener area. The HOG feature descriptor is often used to extract the texture features of an image. The basic idea is to describe the shape and local texture features of the target object by the size and direction of the gray value of the image pixels. The gradient magnitude of the direction generates a histogram as an image feature. Since the HOG feature accumulates the gradient amplitude in a local area, the geometric deformation and optical deformation of the image will not affect its performance.

For a 90×100 size of sub-window image, first use Gamma correction to preprocess the image, with a Gamma coefficient of 1.5; then, calculate the gradient amplitude and gradient angle value of each pixel position of the sub-window image, divide the image into 6×6 pixel cells, and the histogram of 9 channels is used to calculate the gradient amplitude of each cell according to the gradient angle value. Finally, the 3×3 connected interval composed of all cells in the image is used to normalize the statistical value in the interval. The histograms of all connected intervals are connected in series to obtain the HOG features of the sub-window image.

$\chi^{2}$ distance and Bhattacharyya Coefficient (*BC*) [21,22] are two methods commonly used to measure the similarity of histograms. For the two *n*-dimensional normalized histograms x and y, the test formula is defined as follows:

(6) $$\chi^{2}\left(x,y\right)=\sum_{i=1}^{n}\frac{{\left(x_{i}-y_{i}\right)}^{2}}{x_{i}+y_{i}}$$

*BC* is the degree of overlap between two statistical samples. It is also used to measure the similarity of two histograms. The calculation formula of *BC* is as follows:

(7) $$BC\left(x,y\right)=\sum_{i=1}^{n}\sqrt{x_{i}y_{i}}$$

In the formula, n represents the number of partitions of the histogram.

Step 2: Use the nearest neighbor algorithm to calculate the similarity score for each sub-window. After calculating the similarity of each template in the template library and each sub-window, select the top similarity template from high to low, and then calculate the similarity score of each sub-window according to the following formula:

(8) $$S_{similarity}(x_{i})=\frac{1}{K}\sum_{k=1}^{K}\delta_{k}s_{k}(x_{i},t_{k})$$

In the formula, $t_{k}$ represents *k*^th^ template, $s_{k}\left(x_{i},t_{k}\right)$ represents the similarity between the sub-window $x_{i}$ and the template $t_{k}$, $\delta_{k}$ is an indicator function. When $t_{k}$ is a fastener template, $\delta_{k}=1$, and when $t_{k}$ is a background template, $\delta_{k}=0$.

**Figure 4:**
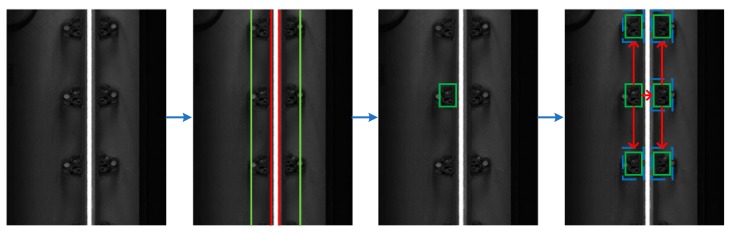
Fastener area positioning results.

Step 3: First, sort all the sub-windows by the similarity score from high to low, select the sub-window with the highest similarity score as the optimal fastener area; then, infer, according to the vertical and horizontal installation distances between the fasteners, the approximate range of other fastener regions. The sub-window with the highest similarity score found within the approximate range is the fastener region. The positioning process of the fastener area is shown in Figure 4. The green rectangle is the fastener area, and the blue dotted rectangle is the approximate range of the other fastener areas.

• Online learning strategies

Because the types of fasteners used on different railway lines or different sections of the same railway line are not uniform, and the background of the image is also very different, it is difficult to completely cover all situations using a fixed number of templates, which can easily lead to problems of false detection and missed detection. Therefore, we propose an online learning strategy for dynamically updating the fastener area template and the background area template during the detection process.

**Figure 5:**
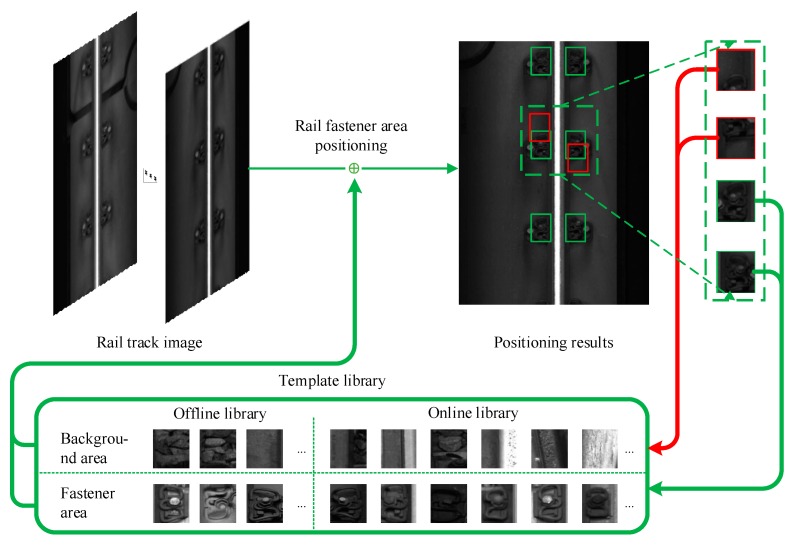
Update process of online template library.

Specifically, the template library is divided into two parts: Online library and offline library. The offline template library contains manually approved fastener templates. The library will not be updated during the detection process, and the online template library will be used during the detection process. The fastener area and background area of the track image of adjacent frames are dynamically updated. The update process of the online template library is shown in Figure 5.

First, add existing templates to the offline template library, including fastener area templates and background area templates. For a railway line to be detected, the user needs to manually locate the fastener area in the first frame of the track image and store it in the offline template library. Then, for each track image to be detected, the track fastener candidate area is detected first, then each fastener area is located according to the template library, and the similarity score is calculated; finally, the online template library is dynamically updated according to the update rule. The rules for updating the online template library are as follows:

(1) Add the fastener area with the highest similarity score on both sides of the rail to the tail of the fastener template queue in the online template library;
(2) grab 2 background areas randomly in the non-fastener areas on both sides of the rail and add them to the tail of the background area template queue in the online template library;
(3) if the length of a queue in the online template library is greater than a preset threshold *L_Max_*, delete the template at the head of the queue.

The online learning strategy is based on the prior knowledge of the track images: The lighting conditions of the track images of adjacent frames, the image background, and the type of fasteners will not change too drastically. According to the update rules, the template at the head of each queue in the online template library is derived from track images with a relatively long interval, and the newly inserted template at the end of the queue is derived from the track images of adjacent frames. Therefore, the lighting conditions, image background, and fastener type of the queue head template may be significantly different from the current track image to be detected. The online learning strategy enables the track fastener area positioning method to adapt to the lighting conditions, image background, and fastener type of the current track image to be detected, which improves the accuracy and generalization of the positioning method.

### 3.3. Locating the Image Region of Nuts or Bolts

**Figure 6:**
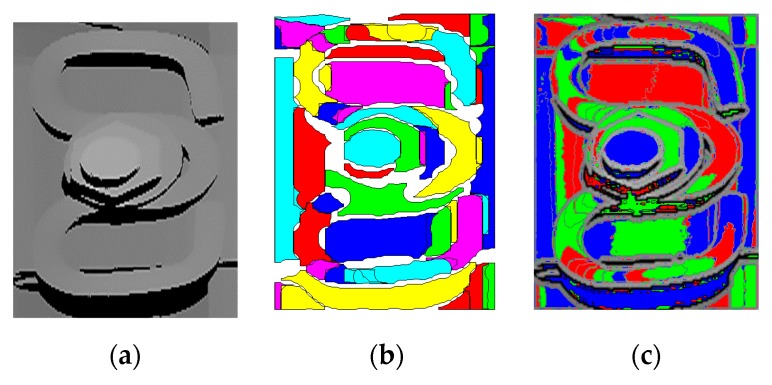

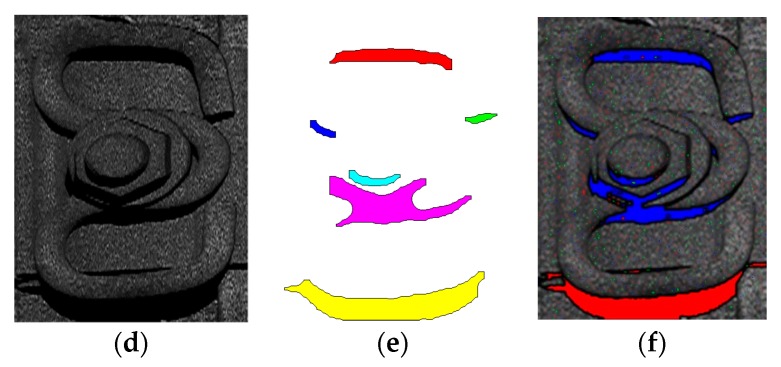
Segmentation results of fastener area in depth map and intensity map. (**a**) Depth map; (**b**) result of area growth method for (**a**); (**c**) result of watershed algorithm for (**a**); (**d**) intensity map; (**b**) result of area growth method for (**b**); (**f**) result of watershed algorithm for (**b**).

After finding the fastener, the next step is positioning the nut or bolt in the fastener area. Take the nut fastening fastener as a typical example (Similarly for bolt fastening fastener), the nut and bolt occupy a large area (about 15%) in the fastener area. At the same time, nuts and bolts have obvious geometric characteristics: Nuts (approximate) are hexagon, bolts (approximate) are round. Therefore, the nuts and bolts can be positioned in the fastener area by image segmentation. Figure 6 shows the segmentation results of the fastener area in the depth map and intensity map (the threshold of the segmentation area size is set to more than 0.1% of the fastener area).

The fastener area of the depth map is relatively smooth, while the fastener area of the intensity map has rich texture features. Therefore, the fastener area of the depth map will achieve better segmentation result. We adopted two widely used image segmentation methods to confirm our previous analysis. 

Based on the results of image segmentation, the nuts and bolts can be determined by further analyzing the geometry and symmetry feature of each segmented image region. For the *n*th sub-region *D’*(*n,x,y*) defined in Equation (3), a binary image *B^(n)^*, in which the element *b_ij_^(n)^* in row *i* and column *j* is generated by:

(9) $$b_{ij}^{\left(n\right)}=\{\begin{array}{c} \begin{array}{cc} 0, & \left(i,j\right)\notin D\prime \left(n,x,y\right) \end{array}\\ \begin{array}{cc} 1, & \left(i,j\right)\in D\prime \left(n,x,y\right) \end{array} \end{array}$$

First, calculate the *n*^th^ segmented sub-region to obtain region area *A^(n)^*, i.e.,

(10) $$A^{\left(n\right)}=\overset{N}{\underset{j}{\sum }}\overset{M}{\underset{i=1}{\sum }}b_{ij}^{\left(n\right)}$$

where *m* and *n* denotes the width and height of the fastener area. Then, we can calculate the centroid of the *n*-th region by

(11) $${\bar{x}}^{\left(n\right)}=\frac{1}{A^{\left(n\right)}}\overset{N}{\underset{j}{\sum }}\overset{M}{\underset{i=1}{\sum }}j\;b_{ij}^{\left(n\right)}$$

(12) $${\bar{y}}^{\left(n\right)}=\frac{1}{A^{\left(n\right)}}\overset{N}{\underset{j}{\sum }}\overset{M}{\underset{i=1}{\sum }}i\;b_{ij}^{\left(n\right)}$$

Further more, we can calculate the “roundness” of region through its second order momentum, *a*^(*n*)^, *b*^(*n*)^ and *c*^(*n*)^, where

(13) $$a^{\left(n\right)}=\frac{1}{A^{\left(n\right)}}\overset{N}{\underset{j}{\sum }}\overset{M}{\underset{i=1}{\sum }}{\left(j-{\bar{x}}^{\left(n\right)}\right)}^{2}b_{ij}^{\left(n\right)}$$

(14) $$b^{\left(n\right)}=\frac{1}{A^{\left(n\right)}}\overset{N}{\underset{j}{\sum }}\overset{M}{\underset{i=1}{\sum }}\left(j-{\bar{x}}^{\left(n\right)}\right)\left(i-{\bar{y}}^{\left(n\right)}\right)\;b_{ij}^{\left(n\right)}$$

(15) $$c^{\left(n\right)}=\frac{1}{A^{\left(n\right)}}\overset{N}{\underset{j}{\sum }}\overset{M}{\underset{i=1}{\sum }}{\left(i-{\bar{y}}^{\left(n\right)}\right)}^{2}\;b_{ij}^{\left(n\right)}$$

The “roundness” of region *n* can be determined by the two eigen-values of the following matrix:

(16) $$R^{\left(n\right)}=\left(\begin{array}{cc} a^{\left(n\right)} & b^{\left(n\right)}\\ b^{\left(n\right)} & a^{\left(n\right)} \end{array}\right)$$

Whose two eigen-values are:

(17) $$\begin{array}{c} \lambda_{1}^{\left(n\right)}=a^{\left(n\right)}+c^{\left(n\right)}-\sqrt{{\left(a^{\left(n\right)}+c^{\left(n\right)}\right)}^{2}-4{\left(b^{\left(n\right)}\right)}^{2}}\\ \lambda_{2}^{\left(n\right)}=a^{\left(n\right)}+c^{\left(n\right)}+\sqrt{{\left(a^{\left(n\right)}+c^{\left(n\right)}\right)}^{2}-4{\left(b^{\left(n\right)}\right)}^{2}} \end{array}$$

The “roundness” of region n can be indicated by the ratio $\tau^{\left(n\right)}$ of the above two eigen-values, i.e.,

(18) $$\tau^{\left(n\right)}=\frac{\lambda_{1}^{\left(n\right)}}{\lambda_{2}^{\left(n\right)}}=\frac{a^{\left(n\right)}+c^{\left(n\right)}-\sqrt{{\left(a^{\left(n\right)}+c^{\left(n\right)}\right)}^{2}-4{\left(b^{\left(n\right)}\right)}^{2}}}{a^{\left(n\right)}+c^{\left(n\right)}+\sqrt{{\left(a^{\left(n\right)}+c^{\left(n\right)}\right)}^{2}-4{\left(b^{\left(n\right)}\right)}^{2}}}$$

The ratio $\tau^{\left(n\right)}$ is in the range between 0 and 1. The smaller $\tau^{\left(n\right)}$ is, the closer to a line the region is. The larger $\tau^{\left(n\right)}$ is, the closer to a round the region is.

By using the above three characters, i.e., region area $A^{\left(n\right)}$, region centroid $\left({\bar{x}}^{\left(n\right)},{\bar{y}}^{\left(n\right)}\right),$ and “roundness” indication $\tau^{\left(n\right)}$, we can find the bolt region in the binary image very accurately through threshold processing. In this paper, the three thresholds are set as following:

$$A^{\left(n\right)}>k_{0}\cdot MN$$

(19) $$k_{1}\cdot N<{\bar{x}}^{\left(n\right)}<k_{2}\cdot N,\;k_{1}\cdot M<{\bar{y}}^{\left(n\right)}<k_{2}\cdot M$$

$$\tau^{\left(n\right)}>k_{3}$$

where $k_{0},\;k_{1},\;k_{2},\;k_{3}\in (0,\;1)$ are the adjustment factor for each filtering threshold, whose value can be assigned according to the fastener type and railway condition in the practical detection. 

Finally, we find the image region of the bolt. The image area of the nut is around the image area of the bolt. By measuring the height of nut and bolt relative to the rail track, we can further determine if the nut is too loose or too tight.

### 3.4. Determining the Tightness of the Fasteners

After determining the image area of the bolt and nut in the depth map, it is necessary to further determine the relative height between the nut and the rail track, so as to further determine whether the nut is too tight or too loose. After the region of bolt and nut is determined, the edge of bolt image and nut image can be obtained by edge detection algorithm. It should be noted that the image of bolt and nut is not “next to each other” due to noise (see Figure 6b). In this paper, we design a fast and stable method to judge the relative height between bolt and rail track. First, according to the area $A^{\left(n\right)}$ of the bolt, the radius of the bolt area can be obtained:

(20) $$r^{\left(n\right)}=\sqrt{A^{\left(n\right)}/\pi }$$

Then, we can find the two opposite edge points of the bolt, i.e.,

(21) $$\left({\bar{x}}^{\left(n\right)}-r^{\left(n\right)},{\bar{y}}^{\left(n\right)}\right)\;\mathrm{and}\;\left({\bar{x}}^{\left(n\right)}+r^{\left(n\right)},{\bar{y}}^{\left(n\right)}\right)$$

Therefore, we roughly locate the position of the bolt, left region $\left({\bar{x}}^{\left(n\right)}-r^{\left(n\right)},{\bar{y}}^{\left(n\right)}\right)$ and right region $\left({\bar{x}}^{\left(n\right)}+r^{\left(n\right)},{\bar{y}}^{\left(n\right)}\right)$. Note that we only focus on the image area on the horizontal straight line of the bolt center, so as to change the nut loose detection from two-dimensional image analysis to one-dimensional signal analysis. Let *h*(*x*, *y*) represent the depth value of point (*x*, *y*) on the depth map. First, we calculate the average value *f* (*x*) in the depth direction of the horizontal “narrow-band area”, which is centered on the horizontal straight line $y={\bar{y}}^{\left(n\right)}$ and has a width of 2ϵ, that is:

(22) $$f\left(x\right)=\frac{1}{2\epsilon }\int_{{\bar{y}}^{\left(n\right)}-\epsilon }^{{\bar{y}}^{\left(n\right)}+\epsilon }h\left(x,y\right)dy$$

**Figure 7:**
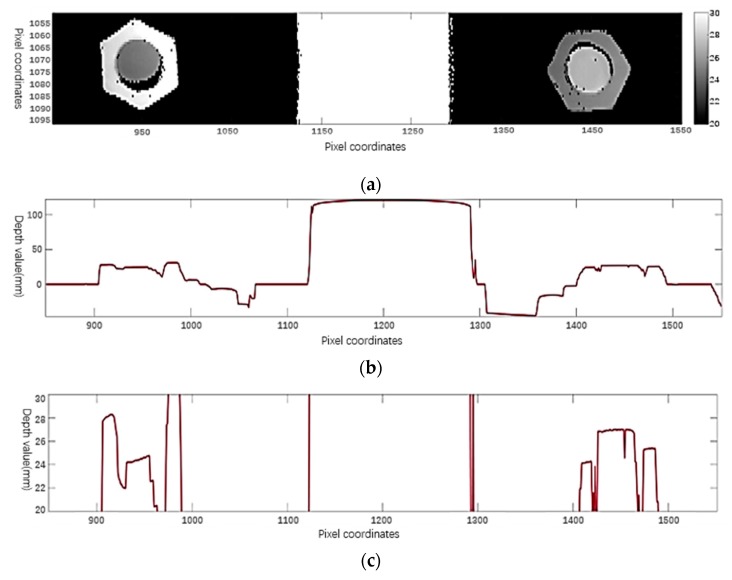
Depth map process: (**a**) Depth map *h* (*x, y*) of fastener area; (**b**) vertical average value *f(x)* across the horizontal “narrow band” area of the fastener; (**c**) partial enlarged display of figure (**b**) (the vertical axis ranges from 20 to 30).

The basic process is shown in Figure 7.

Then, we can judge whether the nut is too loose or too tight by the height difference between the left-edge height of the nut and the left-edge height of the rail head surface. Therefore, we also need to locate the rail in Figure 7b. From Figure 7a,b, we can see that the height of the rail has a large jump on its edge. The rail positioning in curve *f*(*x*) is divided into the following two steps:

Take the derivative of *f*(*x*), and get

(23) $$f^{\prime }\left(x\right)=\frac{d}{dx}f\left(x\right)$$

Generate the trinary function *g*(*x*) using two empirical thresholds $H_{1}$ and $H_{2}$ determined by experimental statistics.

(24) $$g\left(x\right)\{\begin{array}{cc} \begin{array}{cc} 1, & f\prime \left(x\right)>H_{1} \end{array}\\ \begin{array}{cc} -1, & f\prime \left(x\right)<H_{2} \end{array}\\ 0, & \mathrm{otherwise} \end{array}$$

**Figure 8:**
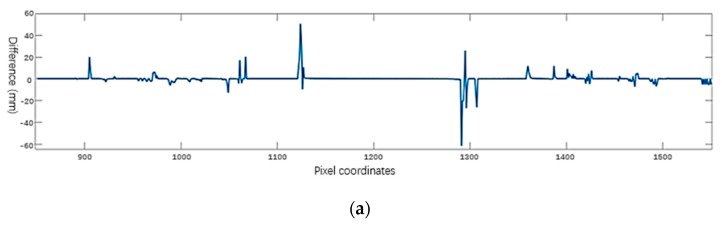

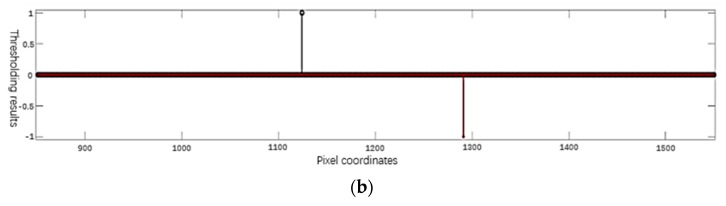
Locate the rail position: (**a**) Derivation *f’(x)* from *f(x)* (or discrete first order difference); (**b**) *g (x)* is obtained by thresholding.

The basic process is shown in Figure 8.

In Figure 8, we accurately locate the position of the rail: 1124 to 1291. The average value from 1261 to 1291 can be selected as the average height of outside wear-free region on the rail head surface, and the result is 120.5990. Furthermore, the height of the nut needs to be determined. According to the prior information of the nut position, the nut is accurately positioned in the image area, e.g.,

(25) $${\bar{x}}^{\left(n\right)}-3r^{\left(n\right)}<x<{\bar{x}}^{\left(n\right)}+3r^{\left(n\right)}$$

**Figure 9:**
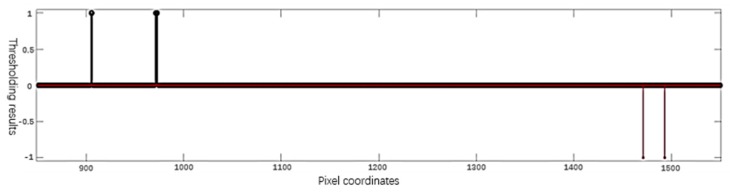
Nut positioning result.

The “region of interest” range for the left nut in Figure 7 is 900 to 1000, and the “region of interest” range for the right nut is 1400 to 1500. Position the leftmost edge of the left nut and the rightmost edge of the right nut. In the corresponding range, the *f’(x)* threshold is processed and located. For example, we can set the threshold value of the left nut to + 5 and the right nut to - 5, and the result is shown in Figure 9:

If there are more than one “non-zero” position in the nut area, for the left nut, select the non-zero position at the leftmost end as $x_{l}$, and for the right nut, select the non-zero position at the rightmost end as $x_{r}$. In Figure 9, the edge position of the left nut is 905, and the edge position of the right nut is 1493. For the left nut, the average value of the interval ($x_{l}+5$,$\;x_{l}+15$) is chosen as the height of the left nut, and the result is 28.1336. The average value of the interval ($x_{r}-15$,$\;x_{r}-5$) is chosen as the height of the right nut, and the result is 24.3395.

Finally, the height difference Δh between the nut height $h_{1}$ and rail surface height $h_{2}$ is compared to determine whether the nut is loose. It is necessary to point out that the nut defects include two situations: Too loose and too tight. If it is too loose, the fastener will be lost easily, else if it is too tight, the clip may be break. Both of these two situations will cause potential safety hazards in railway running. If the difference Δh is greater than the empirical threshold $\xi_{T}$, to give an error warning “too tight”, else if it is less than the empirical threshold $\xi_{L}$, to give an error warning “too loose”. Therefore, the selection of the empirical thresholds $\xi_{T}$ and $\xi_{L}$ is crucial to the defect detection result. Because railway lines are affected by terrain subsidence, fastener types, and construction conditions, it is obviously inappropriate to use the same empirical threshold for the entire line. The threshold is selected here using a dynamic update strategy, that is, the entire line is divided into *N* detection sections with a length of 1 km according to mileage. The threshold is dynamically obtained based on the statistical average of the height difference of the current detection section, and then perform threshold judgment on each fastener in the detection section. The threshold update strategy is as follows:

(26) $$\left\{ \begin{array}{l} \xi_{T}=\frac{1}{i_{e}-i_{b}}\sum_{i=i_{b}}^{i_{e}}\left|h_{i,1}-h_{i,2}\right|+\;\tau_{T}\\ \xi_{L}=\frac{1}{i_{e}-i_{b}}\sum_{i=i_{b}}^{i_{e}}\left|h_{i,1}-h_{i,2}\right|-\tau_{L} \end{array}\right.$$

where $i_{b}$ is the count of the start detection fastener of the current detection section, and $i_{e}$ is the count of the end detection fastener. $\tau_{T}$ and $\tau_{L}$ are two adjustment thresholds, which are a fixed constant determined by experimental evaluation.

## 4. Experiment and Discussion

### 4.1. Laboratory Static M easurement Accuracy

**Figure 10:**
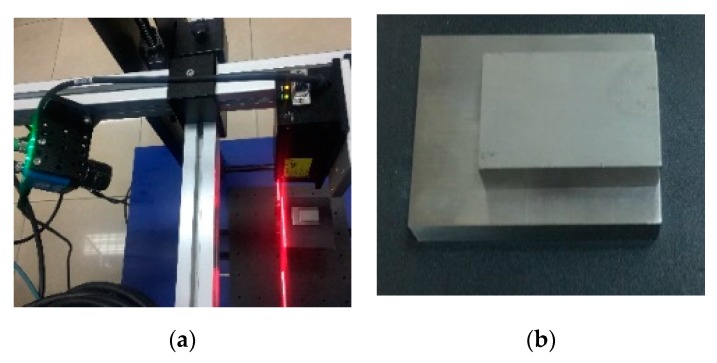

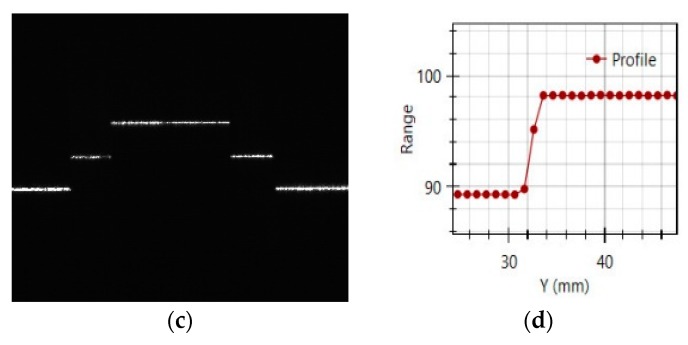
Static measurement precision validation: (**a**) Measuring device; (**b**) two standard blocks; (**c**) profile image of standard block; (**d**) measured profile for standard blocks.

The vision acquisition front end of the 3D measurement component uses a SICK Ranger3 3D camera and an Osela 5 mW line laser with a wavelength of 660 nm, as shown in Figure 10a. The depth measurement range of the 3D measurement component from rail surface to fastener is about 210 mm to 310 mm, of which the end near the 3D measurement component is 210 mm, and the end far from the 3D measurement component is 310 mm. In order to verify the depth measurement accuracy of the component under static conditions, two large and one small metric standard gauge blocks were stacked on the reference table, and the thickness of the standard blocks was 9.00 ± 0.01 mm, as shown in Figure 10b. The 3D measurement component is fixed directly above the standard block, and the light strip image modulated by the standard block is taken. As shown in Figure 10c, the movement of the one-dimensional translation stage below the reference stage (position accuracy 0.05 mm) is achieved Scanning and 3D reconstruction of standard blocks. Calculate the thickness of the uppermost standard block and compare it with the actual thickness of 9.00 mm to get the depth measurement error of the 3D measurement component. The thickness calculation method is as follows: Take any contour line of two standard blocks as shown by the red dashed line in Figure 10b to get the measured profiles of the two standard blocks in Figure 10d, with the red dot in the upper right representing the contour of the upper surface of the small standard block, and the red dot at the lower left indicating the contour of the upper surface of the large standard block. Using the least square method, fit the contour of the upper surface of the small standard block to a straight line, and then calculate the distance from the point on the contour line of the upper surface of the large standard block to the straight line, and record it as *d_i_*, then the standard block’s thickness is

(27) $$d=\frac{1}{n}\sum_{i=1}^{n}d_{i}$$

where *i* is the *i*-th profile point on the upper surface of the large standard block, *n* is the number of profile points.

Because the 3D measurement system has different resolutions at different depth positions, for the accuracy of the test results, move the standard block up and down to obtain the 3D point cloud data of the standard block at 210 mm, 260 mm, and 310 mm, and calculate the standard block’s thickness at three positions as compared with the actual thickness, and three sets of measurement errors are obtained. As shown in Table 1, it can be seen that as the object to be measured moves away from the 3D measurement component, the measurement error gradually increases. In general, the laboratory static measurement accuracy is within 0.1 mm.

### 4.2. On-Site Dynamic Measurement Accuracy

**Figure 11:**
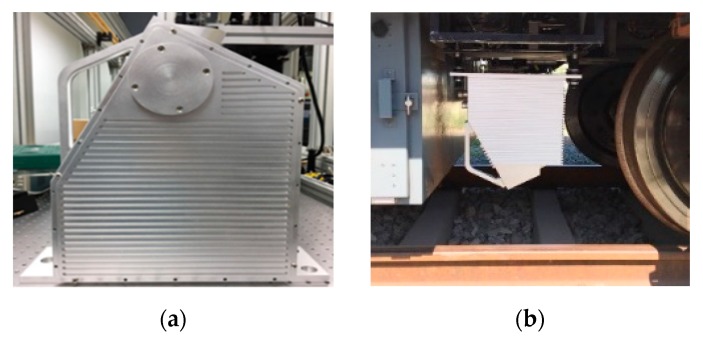

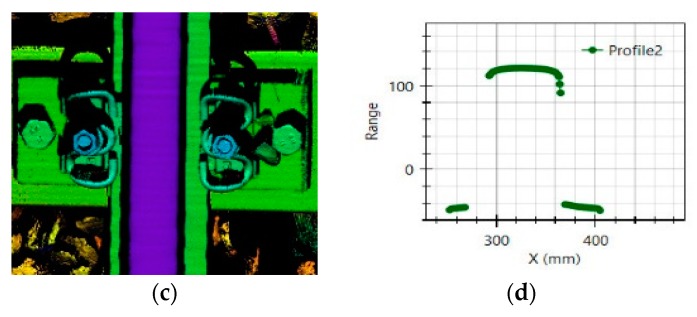
On-site dynamic test: (**a**) 3D measurement components; (**b**) on-site installation; (**c**) rail track 3D reconstruction; (**d**) measured profile of rail.

**Figure 12:**
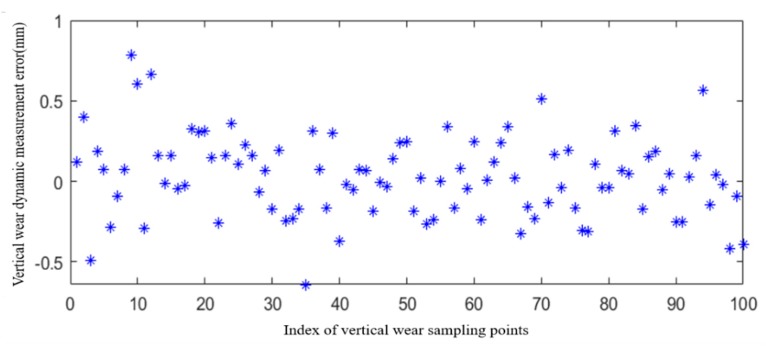
On-site Vertical wear measurement error.

The 3D measurement component is installed on the rail inspection train, and the on-site dynamic test is performed, as shown in Figure 11. Here, the measurement error of rail vertical wear is taken as the dynamic accuracy of the 3D measurement component. Use 3D measurement components to obtain 3D point cloud data of a 100-meter-long rail on site, and calculate the vertical wear of the rail every 1m. Similarly, on-site recheck of rail vertical wear is performed by a rail profiler named “miniprof” (special equipment for measuring rail profile, accuracy up to 0.01 mm) every 1 m, and the dynamic measurement error of the 3D measurement component is defined as the difference between the calculated value of rail vertical wear and the on-site recheck value. Figure 12 shows the vertical measurement error at 100 sampling points. It can be seen that the maximum vertical error is 0.78 mm, the average error is 0.19 mm, and the standard deviation is 0.25 mm.

### 4.3. Fastener Positioning Evaluation

In order to verify that positioning fasteners based on two-dimensional intensity maps is more accurate and reliable, in this experiment, 540 intensity maps and corresponding depth map fasteners were selected as comparative experimental data, and manual labeling was performed to build a track fastener area positioning data set.

Different from ordinary target detection tasks, inaccurate fastener bounding boxes will cause the extracted fastener sub-images to be incomplete, which will cause subsequent fastener recognition methods to produce incorrect recognition results. Therefore, in this experiment, the Intersection-over-Union (IoU) is set to a higher value of 0.9, that is, the IoU value of the positioned bounding box and the labeled true bounding box is greater than 0.9 to be considered valid. IoU is to calculate the coverage of two bounding boxes, which is the ratio of the intersection area to the union area. The calculation method is as follows:

(28) $$IoU=\frac{area\left(DetectionBox\cap GroundTruthBox\right)}{area\left(DetectionBox\cup GroundTruthBox\right)}$$

**Figure 13:**
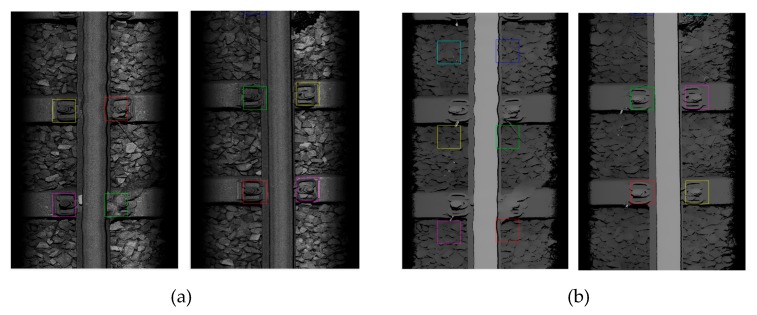
Example of positioning results of rail fastener area: (**a**) Two intensity maps; (**b**) Two depth maps.

The test results show that the accuracy rate of fastener positioning on 540 intensity maps is 537/540, or 99.4%; and the accuracy rate of fastener positioning on the corresponding 540 depth maps is 499/540, or 92.4%. That is to say, the online learning strategy proposed in this paper significantly improves the effectiveness and reliability of the track fastener area positioning method, and the fastener positioning effect based on the two-dimensional intensity map is better. The positioning results of the fastener area of the partial intensity map and depth map are shown in Figure 13.

### 4.4. Nut or Bolt Loose Test

Nuts (bolts) are the key to securing fasteners and are vital to the safety of railway operations. Loose or floating nuts (bolts) can cause fasteners to loosen, which in turn can cause loosening of surrounding fasteners, posing a threat to railway operation safety. This paper proposes a method for detecting loosening of fastener nuts (bolts) based on 3D imaging technology. The specific steps are as follows: First, use 3D measurement components to obtain 3D point cloud data of rails and fasteners. Locate the bolt position through the region of interest ROI to obtain the bolt. Area 3D point cloud, then calculate the bolt height *h*_1_ in this area, and compare it with the average height *h*_2_ of the non-wearing area outside the rail surface.

**Figure 14:**
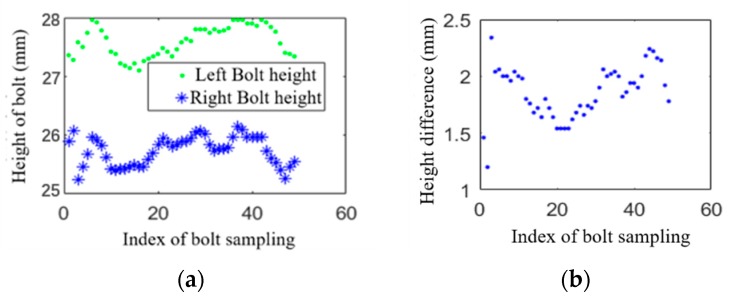
(**a**) Height of left bolt and right bolt; (**b**) height difference between left and right rail bolts.

It should be noted that the rails are not laid horizontally, but there is a small angle inclined toward the inside of the rails, which is called the rail cant. Due to the existence of the rail cant, there is a natural height difference between the nuts (bolts) on the inner and outer sides of the rail. As shown in Figure 14, a number of normal fastener regions are randomly selected on the line, and the height difference between the bolts on the left and right sides of the rail is calculated. It can be observed that the height of the nut (bolt) on the outside of the rail is generally greater than the inside. Based on the actual situation on the site, determine the rail cant as correction term *h*_0_ = 1.9 mm. Therefore, the loosening value of the nut (bolt) can be calculated by the following formula:

(29) $$\Delta h=\left\{ \begin{array}{l} \left|h_{1}-h_{2}\right|,\;inside\;nut\\ \left|h_{1}-h_{2}\right|+h_{0},\;outside\;nut \end{array}\right.$$

Set the nut (bolt) looseness judgment threshold to $\Delta h_{l}$, and over tightening judgment threshold to $\Delta h_{t}$, then the nut (bolt) looseness detection can be determined by the following formula:

(30) $$\left\{ \begin{array}{l} \Delta h>\xi_{T},\;too\;tight\\ \Delta h<\xi_{L},\;too\;loose \end{array}\right.$$

The automatic detection method of rail fastener tightness proposed in this paper is used to analyze the multi-source data collected by the vehicle-mounted equipment. The results of the field test are on a 10-km test line with about 30,000 fasteners, of which 200 anomalies were manually designed as ground truth including 150 bolts loosening and 50 bolts too tight. We evaluate the proposed method by defining precision rate and recall rate as below;

(31) $$\begin{array}{l} Precision=\;TP/(TP+FP);\\ Recall=TP/(TP+FN). \end{array}$$

where *TP* is the number of true positive results; *FP* is the number of false positive results, and *FN* is the number of false negative results. 

Table 2 and Table 3 show the variation of precision and recall with the value of $\tau_{L}$ and $\tau_{T}$ in the range from 1 mm to 10 mm.

A total of 137 fasteners were detected loose, and the recall rate was 91.3% by optimal threshold $\tau_{L}$= 5 mm; 46 too tight fasteners are detected, and the recall rate was 92.0% by optimal threshold $\tau_{T}$ = 6 mm. However, the false positive is a little high due to the many factors such as sunlight interference, terrain changes, and dirty tracks. The error can be reduced by comparing the multiple results from the different detection periods. In fact, it is more meaningful to ensure a high recall with acceptable precision in practical detection application.

## 5. Conclusions

At present, scholars have proposed a variety of algorithms for the processing of 3D visual measurement data, but they are not very versatile, the algorithms are highly correlated with the characteristics of the measured objects, and the related theoretical algorithms are not fully studied. In this context, this article uses the increased third-dimensional data to obtain more information about the measured object, reduce data deviation, improve measurement accuracy, and improve the accuracy of the state evaluation of critical track components. Analysis and research of 3D visual measurement data. We make full use of the two-dimensional intensity and three-dimensional depth multi-source information obtained by the structured light three-dimensional sensor, combine the advantages of the data, and propose an automatic detection algorithm for fasteners and nuts (bolts) combined with multi-source data. Firstly, the position of the fastener is located using the dynamic template matching method from the two-dimensional intensity map, and then the watershed algorithm is used to segment the nut and bolt objects in the corresponding three-dimensional depth map. Finally, based on the location and geometric prior information of the track facility, we designed a model for detecting the tightness of rail fasteners. The experimental results show that the detection method proposed in this paper can effectively detect the abnormal state of the fastener being too loose or too tight, and the detection rate reaches more than 90%, and the automatic detection of the fastener tightness is basically realized.

## Figures and Tables

**Table 1:** Laboratory static measurement precision.

Block Position (mm)	Block Thickness (mm)	Static Measurement Error (mm)
210	8.98	0.02
260	8.94	0.06
310	8.91	0.09

**Table 2:** Variation of precision and recall with the threshold $\tau_{L}$.

$\tau_{L}(mm)$	*TP*	*FP*	*FN*	*Precision*	*Recall*
1	145	17378	5	0.8%	96.7%
2	144	8662	6	1.6%	96.0%
3	141	1991	9	6.6%	94.0%
4	139	108	11	56.3%	92.7%
5	137	41	13	77.0%	91.3%
6	129	29	13	82.5%	86.0%
7	122	15	28	89.1%	81.3%
8	103	13	47	88.8%	68.7%
9	98	10	52	90.7%	65.3%
10	81	10	69	89.0%	54.0%

**Table 3:** Variation of precision and recall with the threshold $\tau_{T}$.

$\tau_{T}(mm)$	*TP*	*FP*	*FN*	*Precision*	*Recall*
1	48	13741	2	0.4%	96.0%
2	48	6266	2	0.8%	96.0%
3	47	1337	3	3.4%	94.0%
4	47	105	3	30.9%	94.0%
5	46	52	4	46.9%	92.0%
6	46	17	4	73.0%	92.0%
7	41	11	9	78.8%	82.0%
8	36	9	14	80.0%	72.0%
9	27	9	23	75.0%	54.0%
10	19	6	27	76.0%	38.0%
